# Comparative organization of the claustrum: what does structure tell us about function?

**DOI:** 10.3389/fnsys.2014.00117

**Published:** 2014-07-02

**Authors:** Joan S. Baizer, Chet C. Sherwood, Michael Noonan, Patrick R. Hof

**Affiliations:** ^1^Department of Physiology and Biophysics, University at BuffaloBuffalo, NY, USA; ^2^The Department of Anthropology, The George Washington UniversityWashington, DC, USA; ^3^Animal Behavior, Ecology and Conservation, Canisius College BuffaloBuffalo, NY, USA; ^4^Fishberg Department of Neuroscience and Friedman Brain Institute, Icahn School of Medicine at Mount SinaiNew York, NY, USA

**Keywords:** gorilla, whale, dolphin, calcium-binding proteins, visual cortex

## Abstract

The claustrum is a subcortical nucleus present in all placental mammals. Many anatomical studies have shown that its inputs are predominantly from the cerebral cortex and its outputs are back to the cortex. This connectivity thus suggests that the claustrum serves to amplify or facilitate information processing in the cerebral cortex. The size and the complexity of the cerebral cortex varies dramatically across species. Some species have lissencephalic brains, with few cortical areas, while others have a greatly expanded cortex and many cortical areas. This evolutionary diversity in the cerebral cortex raises several questions about the claustrum. Does its volume expand in coordination with the expansion of cortex and does it acquire new functions related to the new cortical functions? Here we survey the organization of the claustrum in animals with large brains, including great apes and cetaceans. Our data suggest that the claustrum is not always a continuous structure. In monkeys and gorillas there are a few isolated islands of cells near the main body of the nucleus. In cetaceans, however, there are many isolated cell islands. These data suggest constraints on the possible function of the claustrum. Some authors propose that the claustrum has a more global role in perception or consciousness that requires intraclaustral integration of information. These theories postulate mechanisms like gap junctions between claustral cells or a “syncytium” to mediate intraclaustral processing. The presence of discontinuities in the structure of the claustrum, present but minimal in some primates, but dramatically clear in cetaceans, argues against the proposed mechanisms of intraclaustral processing of information. The best interpretation of function, then, is that each functional subdivision of the claustrum simply contributes to the function of its cortical partner.

The claustrum is a subcortical nucleus described in all placental mammals. Its structure, physiology, connections and neurochemistry have been studied in multiple species (references in Sherk, 1986; Buchanan and Johnson, 2011; Baizer, 2014). Classification schemes vary, but the claustrum is typically divided into two subdivisions, a dorsal and a ventral part, sometimes called the endopiriform nucleus (summary in Baizer, 2014). This paper will focus on the dorsal claustrum. Many studies show that the dorsal claustrum is functionally linked to the cerebral cortex, but the fundamental question of the function of the claustrum is still unanswered. We will consider first what is known about the organization and function of the claustrum from anatomical and electrophysiological studies in the cat and the macaque monkey. We will then ask what the comparative literature on the claustrum suggests about its function. We will supplement existing studies with data on the size and shape of the claustrum in a great ape, the western lowland gorilla, and from two cetaceans, the bottlenose dolphin and the humpback whale. Finally, we will summarize the implications of the comparative neuroanatomy of the claustrum for hypotheses about its function.

## The dorsal claustrum in the cat and the macaque monkey

Many anatomical studies in cat and monkey have shown that the cerebral cortex projects to the claustrum and the claustrum projects back to the cerebral cortex (Druga, 1966, 1968, 1971; Künzle, 1975; Riche and Lanoir, 1978; Turner et al., 1980; LeVay and Sherk, 1981a; Macchi et al., 1981, 1983; Mizuno et al., 1981; Pearson et al., 1982; Bullier et al., 1984; Ungerleider et al., 1984; Weber and Yin, 1984; Kennedy and Bullier, 1985; Guldin et al., 1986; LeVay, 1986; McCourt et al., 1986; Perkel et al., 1986; Sloniewski et al., 1986; Hinova-Palova et al., 1988; Minciacchi et al., 1991, 1995; Boussaoud et al., 1992; Clascá et al., 1992; Morecraft et al., 1992; Baizer et al., 1993, 1997; Tokuno and Tanji, 1993; Tanne-Gariepy et al., 2002; Miyashita et al., 2005; Smith and Alloway, 2010). While there have been suggestions of subcortical connections of the claustrum, data from different studies are inconsistent and contradictory. For example, LeVay and Sherk (1981a), performed extensive experiments using both retrograde and anterograde tracers in the cat, and concluded that “no subcortical projections from the claustrum could be identified”. In contrast, Amaral and Cowan (1980) reported a projection from the claustrum to the hippocampus in the macaque monkey. Similarly, Arikuni and Kubota (1985) reported a projection from claustrum to the caudate nucleus in the macaque monkey, while Saint-Cyr et al. (1990) did not find such a projection. Furthermore, in the most detailed anatomical study of the claustrum LeVay and Sherk (1981a) found that a large injection of retrograde tracer in visual cortex labeled 87% of the cells in the visual claustrum. The unlabeled cells were argued, on the basis of cell size, to represent local interneurons (LeVay and Sherk, 1981a). The weight of the evidence, at present, argues that the major, and possibly only, target of claustral projections is the cortex, and that the major input to the claustrum is from the cortex.

The connections between cortex and claustrum are topographic, with different cortical functional regions connected to dedicated claustrum territories (reviews in Sherk, 1986; Baizer, 2014). The cerebral cortex is characterized by both cytoarchitectural and neurochemical subdivisions, many of which correlate with functional regions (examples and references in Von Bonin and Bailey, 1947; Felleman and Van Essen, 1991; Geyer et al., 1996). However, cytoarchitectural and neurochemical analysis of the claustrum shows structural uniformity with no evidence of structurally- defined subdivisions (LeVay and Sherk, 1981a; Reynhout and Baizer, 1999; Baizer, 2001; Rahman and Baizer, 2007).

**Figure 1:**
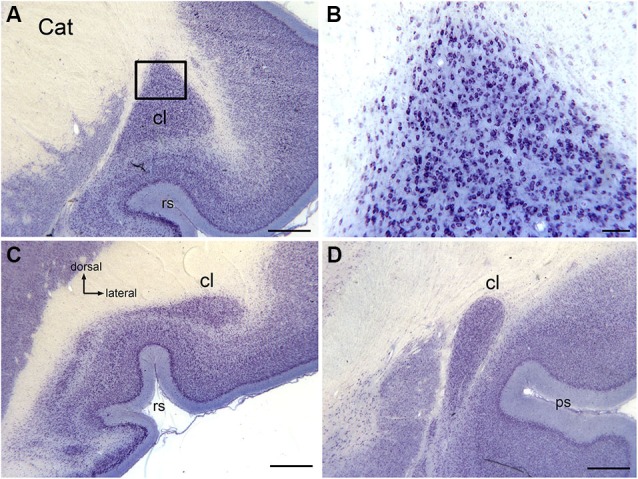
**The claustrum in the cat shown on cresyl violet-stained celloidin-embedded sections. (A)** The claustrum at about its rostro-caudal center. The rectangle shows the location of the image in **(B)**. **(B)** Cells in the claustrum. **(C)** The claustrum at its rostral limit. **(D)** The claustrum at its caudal limit. Scale bars: **A, C, D** = 1 mm; **B** = 100 μm . Abbreviations: cl, claustrum; ps, pseudosylvian sulcus; rs, rhinal sulcus.

The claustrum of the cat extends over about 11 mm rostrocaudally (Snider and Niemer, 1961). Figure 1 illustrates the claustrum of the cat on three sections showing its very different configuration at different rostro-caudal levels. Figure 1A shows a section at about the center of the claustrum, the locus of the large dorsal enlargement in which the visual region is found (LeVay and Sherk, 1981a,b). Figure 1B shows that neurons in the triangular part of the cat claustrum have round or oval somata. Figures 1C,D illustrate the shape of the claustrum at its caudal and rostral limits.

**Figure 2:**
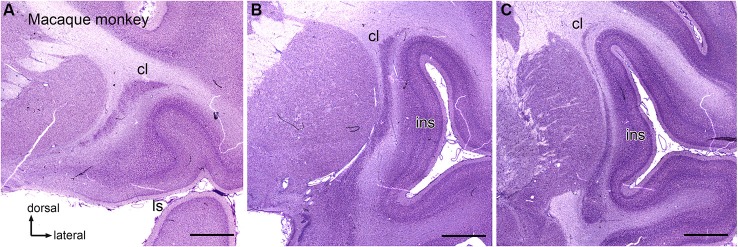
**The claustrum in the macaque monkey. (A)** Rostral claustrum. **(B)** The claustrum at about its rostro-caudal center. **(C)** The caudal claustrum. Note the long, thin vertical stem and the ventral enlargement in **(B)**, **(C)**. Scale bars: **A, B, C** = 2 mm. Abbreviations: cl, claustrum; ls, lateral sulcus; ins, insula.

**Figure 3:**
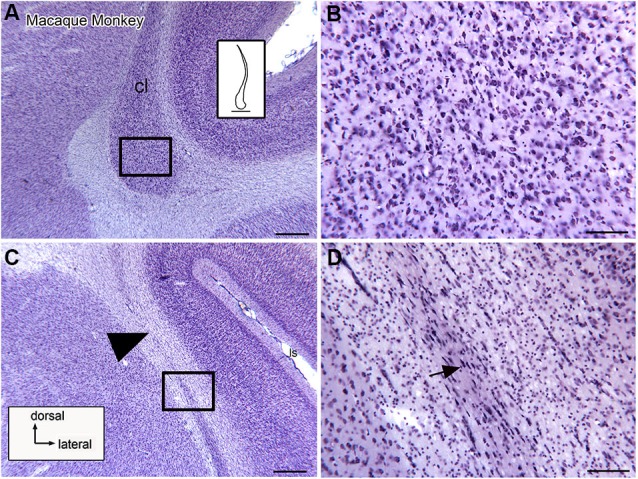
**Neuronal organization of the claustrum in the macaque monkey shown on higher magnification images. (A)** The ventral expansion of the claustrum in the macaque monkey. The rectangle shows the location of the image in **(B)**. The inset shows an outline drawing of the entire claustrum. **(B)** Neurons in the ventral enlargement of the monkey have round or polygonal somata. **(C)** Cells in the long ascending stem of the claustrum. The arrowhead indicates a cell-sparse region. The rectangle shows the location of the image in **(D)**. **(D)** Many neurons in the thin ascending stem have somata that are elongated parallel to the borders of the structure (arrow). Scale bars: **A, C** = 500 μm; **A**, inset = 2 mm; **B, D** = 100 μm.

Anatomical data suggest that there is also a visual region of the macaque monkey claustrum that may contain more than one map of the visual field (Baizer et al., 1993, 1997). Figures 2A–C shows three sections at different rostral-levels of the macaque monkey claustrum. As in the cat, the shape of the claustrum changes with rostro-caudal level. Figure 3 shows the neurons in the claustrum of the macaque monkey at different dorso-ventral levels. Figures 3C,D show that in the long, thin ascending stem of the claustrum many neurons have cell bodies elongated parallel to the long axis of the claustrum (Figure 3D), whereas in wider regions neurons have oval/polymorphic somata (Figure 3B). These differences in soma shape are presumably mirrored by differences in the shapes and extents of dendritic arbors. Further, the long dorsal stem of the claustrum can be discontinuous, with cell sparse regions (Figure 3C, large arrowhead). Interestingly, there is a major difference in the organization of the claustrum between macaque monkey and cat. In both species, the claustrum consists of a long thin stem with an enlargement but this enlargement is dorsally in the cat and ventrally the monkey. In both cat and macaque monkey, the visual claustrum is found within the enlarged region (LeVay and Sherk, 1981a,b; Baizer et al., 1993, 1997).

## Cellular organization of the claustrum

**Figure 4:**
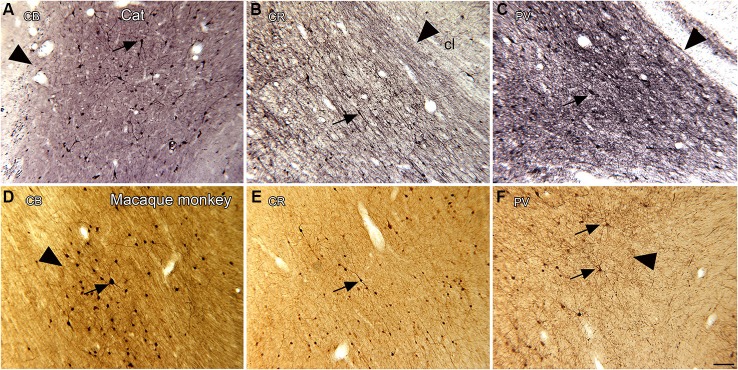
**Spacing and density of neurons in the claustrum of the cat (A, B, C) and macaque monkey (D, E, F) claustrum that are immunoreactive for the calcium-binding proteins. (A)** CB, cat. The arrowhead indicates the medial border of the claustrum; the arrow indicates a labeled cell. **(B)** CR, cat. The arrowhead shows the border of the claustrum and the arrow a cell with a fusiform soma. **(C)** PV, cat. The claustrum is darkly stained; the border (arrowhead) is very clear. The arrow shows a PV-ir neuron. **A, B, C** glucose oxidase modification of DAB for visualization of immunoreactivity. **(D)** CB, monkey. The arrowhead shows the border of the claustrum, the arrow a labeled neuron. **(E)** CR, monkey. The arrow shows a neuron with an elongated soma. **(F)** PV, monkey. The arrowhead shows the edge of the claustrum. The arrows show two large neurons and their dendrites. **D, E, F**, standard DAB visualization of immunoreactivity. Scale bar: **F** = 100 μm; same magnification for all other panels. Abbreviations: CB, calbindin; CR, calretinin; PV, parvalbumin.

Studies in several species identify a population of projection neurons in the claustrum; these neurons are also the targets of descending projections from the neocortex (LeVay and Sherk, 1981a). In addition, there are populations of local interneurons. These were first identified by Golgi impregnation studies (LeVay and Sherk, 1981a; Mamos et al., 1986). Later studies showed that different populations of interneurons are immunoreactive for different calcium-binding proteins (Reynhout and Baizer, 1999; Rahman and Baizer, 2007; Baizer, 2014). Figures 4A–C shows neurons immunoreactive for calbindin (CB; **A**), calretinin (CR, **B**) and parvalbumin (PV, **C**) in the claustrum of the cat. Figures 4D–F show neurons in the claustrum of the macaque monkey immunoreactive for CB **(D)**, CR **(E)** and PV **(F)**. These interneurons provide a substrate for local information processing within the claustrum.

## Theories of the function of the claustrum

There are two very different views of the function of the claustrum. One is that the claustrum is divided into independent functional zones defined by cortical connections and that each zone then influences only its cortical partner. This view derives from anatomical and physiological studies of the visual claustrum of the cat (Olson and Graybiel, 1980; LeVay and Sherk, 1981a,b; Macchi et al., 1981; Sherk and Levay, 1981; Boyapati and Henry, 1985; LeVay, 1986; Updyke, 1993; Minciacchi et al., 1995; Pérez-Cerdá et al., 1996). There is a visuotopic map of the contralateral visual hemifield in the part of the claustrum connected with visual cortex (LeVay and Sherk, 1981a,b; Sherk and Levay, 1981). Neurons in this region have exclusively visual responses and receptive field properties similar to those of cells in V1 (LeVay and Sherk, 1981b; Sherk and Levay, 1981). By this view, it is not possible to define a single function for the claustrum, each functional subdivision would affect the function of its cortical counterpart, which could be sensory or motor or cognitive or affective. Other authors, however, have proposed a more integrative and global role for the claustrum, envisioning that it mediates consciousness (Crick and Koch, 2005) or perceptual integration across sensory modalities (Smythies et al., 2012). These ideas require the integration of information across different functional subdivisions of the claustrum. Anatomical studies did not find evidence for projections among subdivisions of the claustrum (LeVay and Sherk, 1981a), leading to the suggestion of novel mechanisms for intraclaustral information processing. Interneurons in the claustrum are critical for these mechanisms, which include gap junctions among interneurons (Crick and Koch, 2005), and/or dendrodendritic chemical synapses among claustral cells (Crick and Koch, 2005). A related proposal is that the interneurons of the claustrum form an “interactive gap-junction syncytium” (Smythies et al., 2014). These mechanisms require that the claustrum is a continuous structure with neurons in close proximity to each other, that “a densely packed amorphous syncytium [that] constitutes the interior of the claustrum (Smythies et al., 2014). This idea of the interior structure of the claustrum also implies a uniformity of arrangements of dendritic trees and spacing of neurons throughout the claustrum. Examination of the fine structure of the claustrum should allow some evaluation of the plausibility of these mechanisms.

Another implication of the more global theories of claustral function is that the claustrum should increase in importance over evolution. This hypothesis could be examined by quantitative comparative analysis of the relative sizes of claustrum and the cerebral cortex.

## Evolution, the claustrum, and the cortex

Because of the anatomical interdependence of cerebral cortex and claustrum, phylogenetic variation in the organization of the claustrum must be considered in the context of the organization of the cortex. The configuration of the cerebral cortex varies widely among mammals (images of the brains of many different species are shown at http://www.brainmuseum.org). There are many species-specific functional specializations of cortical areas. For example, the maps of the body in sensory and motor cortex adapt to reflect functional specializations in different species, e.g., a representation of the trunk in the somatosensory and motor cortex of the elephant or of high frequency sounds in the auditory cortex of bats (representative studies Edamatsu and Suga, 1993; Fitzpatrick et al., 1993, 1998; Esser et al., 1997; Xiao and Suga, 2004). The size and the complexity of the cerebral cortex change dramatically over evolution. Many rodents are lissencephalic with a relatively small number of cortical areas. By contrast, many anthropoid primates, including humans, have a greatly expanded cerebral cortex with considerable gyrification and an increased number of cortical areas (Agulhon et al., 1998).

**Figure 5:**
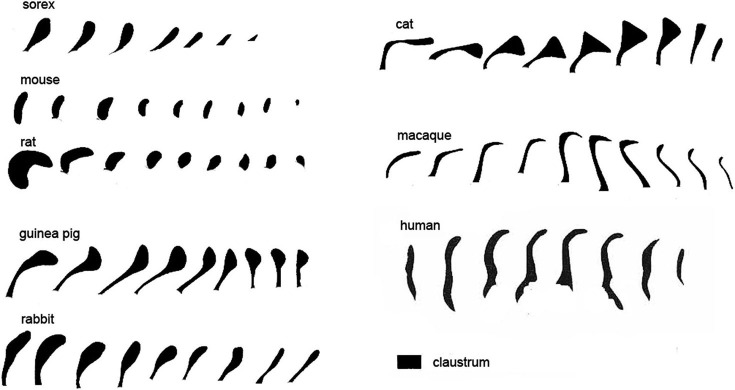
**Variations in the shape of the dorsal claustrum in eight different species**. Modified from Figure 7 in Kowiański et al. (1999). The different drawings are not to scale. Note the presence of a dorsal enlargement and thin stem in sorex, cat and guinea pig compared to the dorsal-ventral symmetry seen in the mouse and the rat and, at some levels, human. In all species the images are arranged with the most rostral on the left.

**Figure 6:**
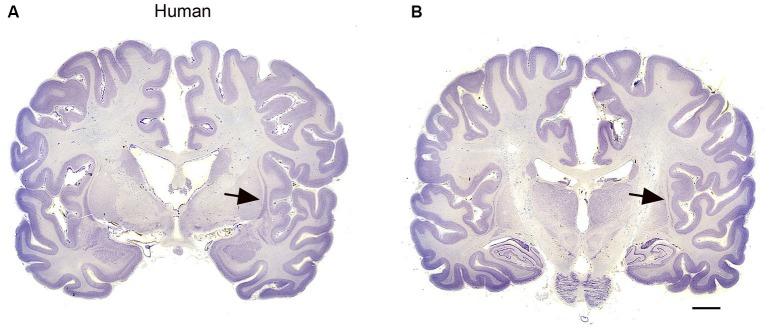
**(A, B)** The claustrum in the human brain. The claustrum is shown on two coronal sections through the human brain about 17 mm apart. **A** is the more rostral. The arrows show the claustrum, which is quite small relative to the total size of the section. The images were downloaded from http://www.brains.rad.msu.edu and, http://brainmuseum.org sites supported by the US National Science Foundation, and used with permission. The brain had been embedded in celloidin and sectioned at 35 μm. Scale bar = 10 mm.

Species differences in cortical organization are mirrored in differences in the size and shape of the claustrum. Buchanan and Johnson (2011) illustrate the shape and size of both cortex and claustrum in 26 different species. Kowiański et al. (1999) illustrated the shape of the claustrum in different species. They recognized five different morphological types; the shape of the dorsal claustrum in 8 different species is illustrated in Figure 5 (modified from Kowiański et al., 1999). It is important to note, however, that while all of these drawings show the claustrum as a continuous structure, examination of Nissl sections from the macaque monkey at higher magnification (as in Figure 3C) shows discontinuities. Figures 6A,B supplement the drawings of the human claustrum with photomicrographs of the human claustrum (at arrows) in Nissl-stained coronal sections of the human brain. The image in A shows a hint of a ventral enlargement as seen in macaque monkey; the image in B supports dorsal-ventral symmetry. A ventral enlargement is present in small-brain lissencephalic New World anthropoid primates such as owl monkeys, squirrel monkeys, titi monkeys, tamarins and marmosets, although it is not as pronounced in the strepsirrhine primates, the lemurs and lorises. However, while illustrations can show species variability in the form of the claustrum, they cannot relate the differences in the shape and size of the claustrum to differences in the numbers or organization or specializations of cortical areas.

In addition to illustrating the species differences in the appearance of the claustrum, Kowiański et al. (1999) asked a critical question: do the claustrum and the cerebral cortex expand in parallel? They calculated the ratio of the volume of the claustrum to the volume of the cerebral hemispheres in several species; the data showed that the volume of the claustrum does not increase proportionately at the same rate to the volume of the cerebral cortex (Kowiański et al., 1999). Of the species they examined, the ratio of the volume of the claustrum to the volume of the isocortex was highest in mice (6.5%) and lowest in humans (0.45%). These data suggest that as the cerebral cortex expands the relative size of the claustrum actually decreases.

Both the qualitative and quantitative analyses of the claustrum in human raise questions about the similarity of its organization to that of other species. The shape of the claustrum in the human is strikingly different from that of the rodents, the cat and most primate species. Does the claustrum in human connect only with the “older” cortical structures, i.e., the primary sensory and motor areas or are there parts of the claustrum that are more related to association cortical areas and their functions? Is the claustrum in humans structurally uniform as it is in animals? In humans, there is lateralization of both language and handedness and this lateralization is also mirrored by structural asymmetries (Steinmetz et al., 1989; Jäncke et al., 1994; Steinmetz, 1996; Westbury et al., 1999; Pujol et al., 2002). Might this lateralization be reflected in left-right asymmetries in the claustrum? Another characteristic of cerebral cortex in humans, great apes and other primates with gyrified brains is individual variability in the pattern of cortical sulci and gyri (Steinmetz, 1996; Westbury et al., 1999). Is this individual variability mirrored in individual differences in claustrum morphology?

There are thus many questions about the organization and function of the claustrum in humans. How may these questions be addressed? Direct studies of connections and function using invasive techniques cannot be undertaken in humans or other great apes. However, descriptive anatomy based on postmortem tissue, as well as imaging studies, are powerful techniques that can be used in human.

There are studies of the cell types in the human claustrum (Braak and Braak, 1982; Spahn and Braak, 1985; Hinova-Palova et al., 2013). The anatomical data in general support the idea of the presence of both projection neurons and interneurons, and overall structural uniformity within the claustrum.

There are also imaging studies of the human claustrum using a variety of techniques. One study suggests that the claustrum in humans, as in other species, is interconnected with many regions of the cerebral cortex (Milardi et al., 2013). That study also showed both individual variability and left-right differences in claustral volumes, in keeping with findings for the human cerebral cortex. There were also sex differences in claustral volume, with males having a larger volume than females. This may reflect overall sex differences in brain size (Leonard et al., 2008; Luders et al., 2009) (One caution about the results of this study: a pathway between claustrum and basal ganglia was also found, such a pathway was not described in the most detailed experimental study of the connections of the claustrum (LeVay and Sherk, 1981a)).

There are many imaging studies that suggest a role of the claustrum in rather diverse functions. At this point, these studies must be interpreted with caution, as the imaging resolution may not yet be sufficient to distinguish activation of the claustrum, which in humans is very thin, from that in surrounding brain regions like the cerebral cortex and the putamen. Imaging resolution is certainly not yet able to resolve activation differences within different functional zones of the claustrum itself. For example, Wegiel et al. (2014, p. 227) list 10 functions in which the claustrum has been implicated including “experiential dread” and “suppression of natural urges”. In addition to the studies listed by Wegiel et al. (2014) there is a PET imaging study that suggested a role for the claustrum in sexual function (Redouté et al., 2000; Figure 3), an fMRI study linked the claustrum to ADHD (Wang et al., 2013), another to aesthetic judgment (Ishizu and Zeki, 2013) and yet another suggested pathology of the claustrum in bipolar disorder (Selvaraj et al., 2012). Increased resolution of imaging techniques may clarify the role of the claustrum in these, and other, as yet unstudied, functions.

## The claustrum in the gorilla

**Figure 7:**
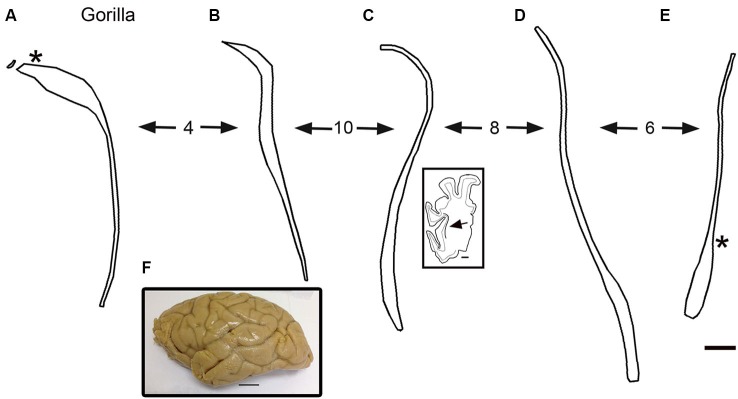
**(A–E)** Outline drawings of the claustrum of the gorilla from five coronal cresyl-violet stained sections. **A** is the most rostral. The spacing of the sections (mm) is indicated by the numbers between the arrows. **(A)** The asterisk shows the approximate location of the image in Figures 8A,C. The inset shows an outline drawing of the left half of the cerebral cortex on the section for the level of the claustrum shown; the arrow indicates the location of the claustrum on this section. **(E)** The asterisk shows the approximate location of the image in Figure 8C. **(F)** Lateral view of the left hemisphere of a gorilla showing the sulci and gyri. Caudal is to the right. Scale bars: **E** = 2 mm, same scale for **A–D**; **C**, inset = 5 mm; **F** = 2 cm.

**Figure 8:**
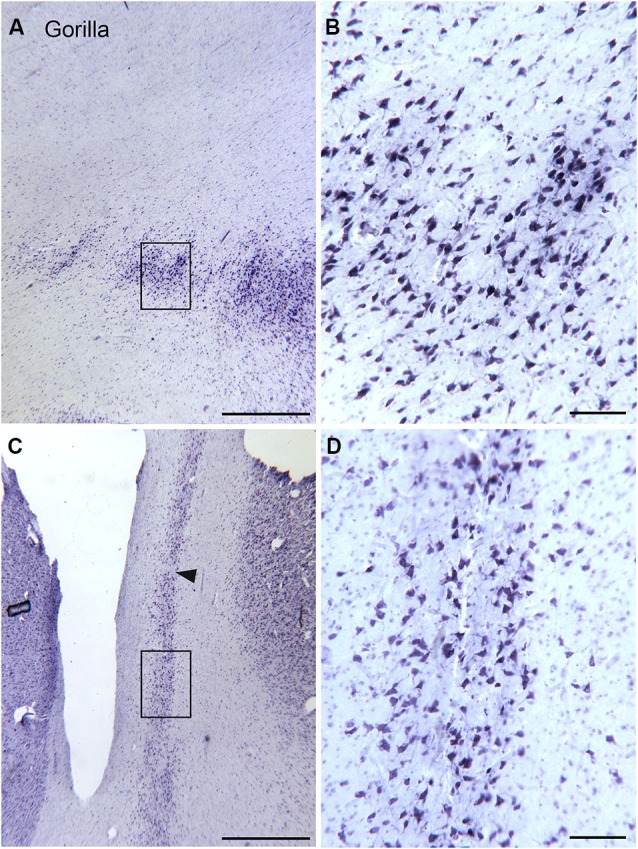
**Size shape and density of neurons in the claustrum of the gorilla at different rostro-caudal and dorso-ventral levels. (A)** Cell islands in the dorsal claustrum (section outlined on Figure 7A). The rectangle shows the location of the image in **(B)**. **(B)** Stained somata in the claustrum; many somata are multipolar. Scale bar = 100 μm. **(C)** Image of the more ventral and caudal claustrum; the section was outlined in Figure 7E. The rectangle shows the location of the image in **(D)**. The arrowhead indicates a cell-sparse region. **(D)** Somata in the ventral and caudal claustrum; many are multipolar. Scale bars: **A, C** = 1 mm; **B, D** = 100 μm.

The drawings in Figure 5 suggest that the human claustrum has more dorsal-ventral symmetry than seen in other species. In order to see if this is unique to humans or is a general feature of the brains of great apes, we examined the claustrum in the gorilla. Figure 7F shows a lateral view of the gorilla brain; its rostro-caudal extent is about 130 mm. There is a more complex pattern of sulci and gyri than seen in the macaque monkey. Examination of cresyl violet-stained sections showed that the claustrum was present over about 33 mm in the rostro-caudal direction. Figures 7A–E show five outline drawings of the claustrum at different rostro-caudal levels. The claustrum is very elongated in the dorso-ventral direction, extending as far as 20 mm (Figure 7D); it is also very thin. Comparison of the claustrum in the gorilla (Figure 7) and the human (Figures 5, 6) shows that the claustrum in the gorilla is much longer and narrower than in the human. Strikingly, at some levels, the claustrum is not a continuous structure; Figure 7A shows an isolated cluster of neurons in the dorsal part of the claustrum. Figures 8A,C show lower magnification photomicrographs of the claustrum on two different sections, one at a more dorsal level and the other at a more ventral level. Again the isolated clusters of cells are apparent (Figure 8A). The shapes of the somata also vary with location in the claustrum. Figures 8B,D shows higher magnification images showing the numbers, shape, and density of stained neurons. Figure 8B shows somata that are elongated roughly parallel to the dorso-ventral axis. Figure 8D shows larger stained somata that are polygonal with no preferred orientation. These differences in soma shape probably correlate with differences in the shape and extent of the dendritic trees, differences not considered in the “gap junction syncytium” hypothesis. Since the brains for the cat and monkey were not prepared in the same way (celloidin vs. frozen sections) meaningful comparisons of neuronal size or neuronal packing density among these species cannot be made.

## The claustrum in cetaceans

**Figure 9:**
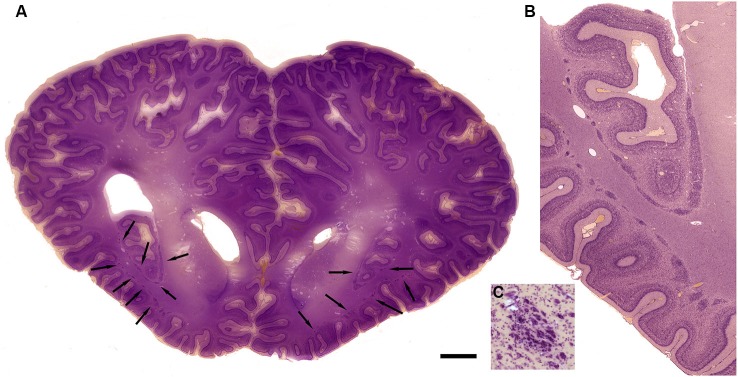
**Morphology of the claustrum in the bottlenose dolphin (*Tursiops truncatus*). (A)** Coronal section through the brain of a 4 year old *Tursiops*. The arrows point to the location of the claustrum around the anterior portion of the insular pocket and to the highly unusual distribution of many claustral island along cortical gyri in the prefrontal cortex, shown at higher magnification in **(B)**. **(C)** Cellular details of one island of claustral neurons in the anterior portion of the ectosylvian gyrus. Scale bars = 1 cm **(A)**, 4 mm **(B)**, and 100 μm **(C)**.

**Figure 10:**
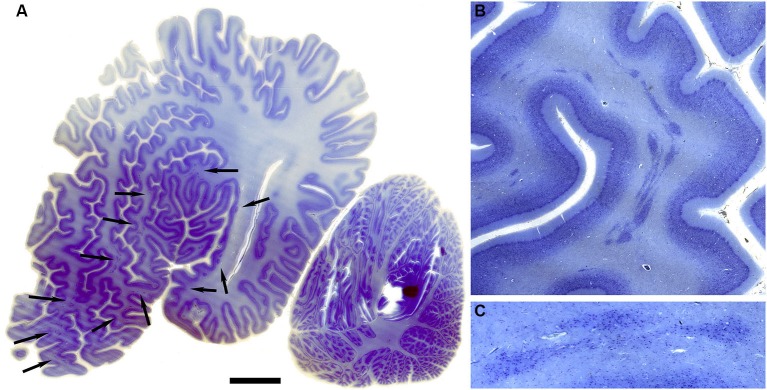
**Morphology of the claustrum in the humpback whale (*Megaptera novaeangliae*). (A)** Parasagittal section through one hemisphere of an adult humpback whale. There are a very large number of claustral islands dispersed in the white matter underlying the perisylvian, ectosylvian, and suparsylvian cortex, as well as in the frontal pole (arrows). **(B)** Higher magnification image of a large cluster of these claustral neurons in the white matter of the suprasylvian gyrus. **(C)** Shows cellular details. Note that as in the bottlenose dolphin, these claustral island are completely separate from the neocortex. Scale bars = 2 cm **(A)**, 6 mm **(B)**, and 1.5 mm **(C)**.

The drawings in Figure 5 do not include all of the forms that the claustrum can take. In the elephant, the claustrum is elongated toward the frontal pole of the cortex and appears as cell islands just under the cortex (see Figure 2 in Hakeem et al., 2009). Cell islands are also found in cetaceans, animals with well developed and highly folded cerebral cortices. Figure 9A shows the highly folded cortex in the brain of the bottlenose dolphin; arrows indicate the locations of cell islands comprising the claustrum. Figure 9B shows claustral islands at higher magnification and Figure 9C shows the cellular organization of one such island. It is not clear from the anatomy, of course, if the islands represent fragmented functional subdivisions (as would be the case, for example, if the the visual claustrum of the cat were broken up into isolated cell clusters) or if each island is a self-contained unit. Figure 10A shows the highly folded cerebral cortex of the whale on a parasagittal section; the arrows show the very scattered islands of claustral cells. Figures 1B,C show these cell clusters in more detail.

## Conclusions: what is the function of the claustrum?

The comparative data summarized here argue against the hypothesis that the claustrum has an important role in global perceptual integration and/or consciousness. First, examination of the shape of the claustrum in different species shows considerable variability, with several species having very long, thin claustral “stems” that would seem to impose constraints on the proposed mechanisms of intraclaustral processing. These notions of intraclaustral processing also require that the claustrum is a continuous structure, and that clearly is not the case in a number of species. We have shown sections in both macaque monkey and gorilla in which the claustrum is characterized by separated cell clusters. Claustral fragmentation is seen even more dramatically in the bottlenose dolphin and humpback whale. Another argument against a major integrative role for the claustrum, however, comes from the quantitative analysis showing its decreased relative size (as indicated by the ratio of volumes) as cerebral cortex expands (Kowiański et al., 1999). Rather than suggesting a global role for the claustrum, the comparative data reinforce the early view that each functional division of the claustrum is independent from the others, and works with the cortical area with which it is interconnected. By this view, the function of each subdivision would be to enhance or modulate the function of its cortical partner. Thus, the anatomical data do not support a more global role of the claustrum. The fact that expansion of the claustrum does not parallel cortical expansion suggests that the function of amplifying or adjusting cortical excitability was of greater importance in lissencephalic animals, and that the importance of claustrum for cortical operations has decreased as cortical complexity has increased.

## Materials and methods

### Tissue and histology

#### Cat, macaque monkey

We photographed archival celloidin-embedded cresyl violet stained sections of the cat and macaque monkey claustrum. These slides had been prepared in the laboratory of Dr. Mitchell Glickstein, then at Brown University. We also photographed archival immunostained sections that had been prepared in the course of earlier studies (Reynhout and Baizer, 1999; Rahman and Baizer, 2007).

#### Gorillas

We obtained the brains of two western lowland gorillas from the Buffalo Zoo. Gorilla 1 was a female, age at death 6 years, cause of death unknown. Gorilla 2 was a male, age at death 20 years; the cause of death was cancer. The brains were removed and stored in 10% formalin. No data on the postmortem intervals of this tissue were available. The brains were cryoprotected in 15% then 30% sucrose in 10% formalin. The brain of Gorilla 1 was blocked to include the claustrum; the brain of Gorilla 2 was divided into four equal size blocks. Frozen sections 40 μm thick were cut in the coronal plane on an American Optical sliding microtome fitted with a custom-built copper freezing platform. All sections were collected and stored in large plastic compartment boxes, 5 sections/compartment in 5% formalin in a cold room. Series of sections 2 mm apart were mounted onto gelled slides (Brain Research Laboratories, Newton, MA) and stained for Nissl substance with cresyl violet (following the protocol of LaBossiere and Glickstein, 1976).

#### Cetaceans

We photographed archival celloidin-embedded cresyl-violet stained sections of a bottlenose dolphin brain that had been used in earlier projects (Hof et al., 2005). We also photographed slides of the brain of a stranded humpback whale; the cerebral cortex of that animal was described in an earlier report (Hof and Van der Gucht, 2007).

#### Outline drawings

The outlines of the gorilla claustrum in Figure 7 were drawn from sections using MDplot software (Accustage, Shoreview, MN) with stage encoders mounted on a Leitz Dialux 20 microscope. These drawings were saved as .bmp files using the screen capture function of the software and then assembled into figures with Adobe Photoshop (Adobe, San Jose, CA).

#### Photography

Images (1600 × 1200 pixels) were captured with a SPOT Insight Color Mosaic camera (Diagnostic Imaging, Sterling Heights, MI) mounted on the Leitz microscope. We used Adobe Photoshop to adjust the brightness and contrast of images and to assemble and label the figures.

## Conflict of interest statement

The authors declare that the research was conducted in the absence of any commercial or financial relationships that could be construed as a potential conflict of interest.
